# 

**Published:** 1833-10

**Authors:** 


LONDON:

J. SOUTER, 73, ST. PAUL'S CHURCH-YARD;

SOLD ALSO BY BURGESS AND HILL; J. CHURCHILL; E. COX; J. HIGHLEY;

RENSHAW AND RUSH, &c.

W. JACKSON, NEW YORK, AGENT FOR THE UNITED STATES.

1834.

J. AND C. ADLARD, PRINTERS,
BARTHOLOMEW CLOSE.

### NOTICES.

*We regret that we cannot insert* Dr. JAMESON'S *communication: he can obtain it at our publisher's.*

*We will shortly answer* Dr. AITON'S *letter.*

Dr. ROUPELL'S "*Illustrations of the Effects of Poisons*" *came too late for review in this number; as did* Dr. CLANNY'S *work entitled* "*Hyperanthroxis.*"

